# A FOXM1 Dependent Mesenchymal-Epithelial Transition in Retinal Pigment Epithelium Cells

**DOI:** 10.1371/journal.pone.0130379

**Published:** 2015-06-29

**Authors:** Parul Choudhary, Benjamin Thomas Dodsworth, Ben Sidders, Alex Gutteridge, Christos Michaelides, Joshua Kane Duckworth, Paul John Whiting, Caroline Louise Benn

**Affiliations:** Neusentis, Pfizer Ltd, The Portway, Granta Park, Great Abington, Cambridge, United Kingdom; Wenzhou Medical University, CHINA

## Abstract

The integrity of the epithelium is maintained by a complex but regulated interplay of processes that allow conversion of a proliferative state into a stably differentiated state. In this study, using human embryonic stem cell (hESC) derived Retinal Pigment Epithelium (RPE) cells as a model; we have investigated the molecular mechanisms that affect attainment of the epithelial phenotype. We demonstrate that RPE undergo a Mesenchymal–Epithelial Transition in culture before acquiring an epithelial phenotype in a FOXM1 dependent manner. We show that FOXM1 directly regulates proliferation of RPE through transcriptional control of cell cycle associated genes. Additionally, FOXM1 modulates expression of the signaling ligands BMP7 and Wnt5B which act reciprocally to enable epithelialization. This data uncovers a novel effect of FOXM1 dependent activities in contributing towards epithelial fate acquisition and furthers our understanding of the molecular regulators of a cell type that is currently being evaluated as a cell therapy.

## Introduction

Retinal Pigment Epithelium (RPE) cells are cobblestone shaped, pigmented cells situated as a tightly packed monolayer behind the photoreceptors in the retina. RPE are polarised, having differential localization of proteins at their apical and basal surfaces, and perform several functions such as metabolism and storage of retinoid, phagocytosis of rod outer segments, absorption of scattered light, barrier activity and ion transport [1]. This helps to maintain homeostasis in the retina and contributes to the complex process of vision. Loss of RPE function manifests itself in diseases such as Age-Related Macular Degeneration, Retinitis Pigmentosa, Best’s disease, diabetic retinopathies among others, which often result in loss of vision [2]. A potential treatment for at least some of these conditions is replacement of the dysfunctional RPE with a healthy epithelium. Human embryonic stem cells (hESC) offer a prospective limitless source of material to derive healthy RPE suitable for transplantation, which is an attractive therapeutic option.

Several studies have demonstrated successful derivation of mature RPE from different hESC and induced pluripotent stem (iPS) cell lines using a spontaneous differentiation method which produces RPE, albeit at low efficiency, on prolonged culture [3–7]. Attempts have been made to direct the differentiation of hESC towards the RPE lineage by supplementation of growth media with soluble factors and small molecules which have helped to increase yield and decrease variability as well as culture period [8–11]. Clusters of RPE derived from hESC can be manually dissected and cultured by the ‘outgrowth method’ where RPE present at the periphery of the cluster or sheet proliferate and migrate leading to an expansion of the seed culture [12,13]. Another approach is the enzymatic dissociation of the pigmented RPE clusters into single cells which can then be plated down on extracellular matrix (ECM) coated surfaces and expanded by proliferation [3,14]. The latter approach has been used in our study.

hESC-derived RPE have been shown to be equivalent to primary RPE at the transcriptional and functional level [15–17] and their transplantation has long-term protective effects leading to restored visual function in animal models of retinal dystrophy [18–20]. In particular, detailed studies on RPE derived from the SHEF1.3 hESC line have shown them to be phenotypically, molecularly and functionally equivalent to native RPE [3]. However, use of RPE for cell replacement therapy in current clinical approaches requires *in vitro* expansion of a relatively small population of RPE cells that are generated from hESC. Therefore, it is important to gain in-depth understanding of the transitions that occur in these cells during culture and of the transcriptional regulators and signalling pathways that are involved in this process.

In this report, we show that RPE, when dissociated and cultured, lose their epithelial characteristics and instead uptake a de-differentiated mesenchymal phenotype. This is followed by a mesenchymal-epithelial transition (MET) where cells revert to the epithelial state. The process of MET has been shown to be important in diverse events such as cellular reprogramming, organ development and metastasis [21–23]. A key feature of a canonical MET is the downregulation of N-Cadherin (CDH2) concomitant with the upregulation of E-Cadherin (CDH1) which imparts epithelial characteristics to cells. This is attributed to downregulation of EMT-inducing transcription factors (EMT-TFs) such as Snail, Slug, ZEB1/2, TWIST, GSC and others which have been extensively described to induce a classical epithelial-mesenchymal transition during cancer and fibrosis [24,25]. These EMT-TFs play a central role in repression of E-Cadherin in mesenchymal cells and their downregulation is accompanied by a reversion of the mesenchymal to the epithelial state.

Our study shows that RPE culture displays phenotypic and molecular changes expected with a mesenchymal-epithelial transition. However, it does not appear to be regulated by classical EMT-TFs implicated in examples of METs described in other cellular systems. Instead, we show that the transcription factor FOXM1 (Forkhead box M1) plays an important role in RPE MET and acquisition of the epithelial fate. FOXM1 belongs to a family of evolutionarily conserved transcriptional regulators defined by a common DNA-binding domain known as the winged-helix domain [26]. It is a key mediator of cell cycle progression and regulates the G1-S and G2-M phase transitions [27,28]. Loss of *Foxm1* in mice results in embryonic lethality owing to inability to undergo mitosis [29]. Accumulating evidence suggests that FOXM1 functions as a proto-oncogene and contributes to the initiation and progression of many types of cancers of the breast, liver, lung, brain and prostate [30]. However, compared to what is known about the role of FOXM1 in cancer, its function in a physiological epithelial system remains to be fully understood. Here we demonstrate that FOXM1 positively regulates MET and is required for acquisition of epithelial phenotype in RPE. This is achieved through direct regulation of proliferation and targeting of cell cycle associated genes. We also show that FOXM1 modulates the levels of BMP and Wnt signalling ligands, which as we demonstrate, are important signalling pathways for achieving MET. A coordinated interplay between these distinct FOXM1-dependent functions is required to remodel RPE into a stable epithelial state following proliferation.

## Materials and Methods

### Cell culture and manipulations

RPE were generated from the hESC line SHEF1 (obtained from Axordia Ltd, also available in the UK Stem Cell Bank with the accession number R-05-007) or iPSC reprogrammed from blood obtained from healthy volunteers from NHSBT using the CytoTune-iPS Reprogramming kit (Life Technologies). ARPE19 cell line was obtained from ATCC. RPE were generated by the spontaneous differentiation method described previously [12]. Pluripotent cells were cultured as feeder-free colonies on hESC-qualified Matrigel (BD) in mTesR1 (StemCell Technologies) media. RPE foci were excised with a scalpel and dissociated into a single cell suspension using Accutase (Gibco) and plated onto CellStart (Invitrogen) coated surfaces. Where required, media was supplemented with recombinant human Wnt5B (500ng/ml; R&D Systems), BMP-4/7 (75ng/ml; R&D Systems), Thiostrepton (Sigma), LDN-193189 (10μM; Stemgent), WAY-262611 (10μM; Enzo Lifesciences). Fresh factors were replenished on alternate days. Knockdown of targets of interest was carried out using predesigned siRNA ON TARGET plus Smartpools (Dharmacon; FOXM1: L-009762-00, SNAIL: L-010847-01, SLUG: L-017386-00, ZEB1: L-006564-01, TWIST1: L-006434-00, GSC: L-019261-01, GAPDH: L-004253-00, Non targeting control: D-001810-01) with the DharmaFect1 reagent (Dharmacon) according to manufacturer’s instructions. The FOXM1 overexpression vector was purchased from Origene (SC112825) and transfected into RPE using the Effectene reagent (Qiagen) according to manufacturer’s instructions, although some effects of reagent toxicity were noted. For the wound closure assay, a scratch was introduced in a confluent RPE monolayer grown in 96 well plates, using the LEAP instrument. Bright field images were captured on the ImageXpress platform (Molecular devices) and width of the scratch was measured manually using the MetaXpress 3.1 software (Molecular devices).

### RNA extraction, cDNA synthesis and quantitative PCR

Total RNA was extracted from RPE cells using the RNEasy Mini or Micro Kit (Qiagen) with on-column DNase digestion. cDNA was synthesized using the High Capacity cDNA Synthesis kit (Applied Biosystems). Individual gene expression was assessed using predesigned Taqman assays (Applied Biosystems) and the reactions were carried out on the CFX96 iCycler platform (Biorad). Gene expression in all instances was quantified by the 2^-ΔΔCt^ relative quantification method [31] and normalized to geometric means of at least two housekeeping genes.

### Microarray analysis

mRNA was hybridized on Illumina HT-12v4 BeadChips according to manufacturer’s instructions. The microarray data are available in the ArrayExpress database under accession number E-MTAB-854.

### Immunostaining

Samples were fixed in 4% paraformaldehyde in PBS for 15 min followed by blocking and permeabilization using 0.3% Triton X-100 in PBS and 10% normal donkey serum (NDS). Primary antibodies used in this study are: mouse anti-PMEL17 (1:25, Dako M0634), rabbit anti-Ki67 (1:500, VectorLabs VP-K451), rabbit anti-ZO1 (1:100, Zymed 187430), mouse anti-αSMA (1:1000, Sigma A5228), mouse anti-FOXM1 (1:50, Abcam ab55006), mouse anti-CRALBP (1:200, Affinity Biosciences MA1-813). For measurement of EdU incorporation, cells were treated with 10μM EdU 18 hours prior to fixation. EdU incorporation was measured by using the Click-iT 488 Imaging kit (Life Technologies) according to manufacturer’s instructions. Nuclei were counterstained with the nuclear dye Hoechst. All images were captured and analysed on the ImageXpress platform (Molecular devices).

### Chromatin immunoprecipitation, sequencing and analysis

ChIP was performed essentially as described before [32]. 1 million RPE cells (collected at Day 5 of culture, when FOXM1 expression levels are high) were crosslinked with 1% Formaldehyde for 10 minutes and the reaction was quenched with 125mM glycine. Sonication was performed on isolated nuclei using the Bioruptor Plus with cooling system for 35 cycles of each 30 seconds on and 30 seconds off at the maximum setting leading to a fragment size between 200–300bp. For each IP, 50μl of Protein A Dynabeads were incubated with 5μg of the appropriate antibody [anti-FOXM1 (Santa Cruz Biotechnology, SC-502) or Rabbit IgG (VectorLabs, I-1000)] overnight at 4°C. Chromatin immunoprecipitation was performed by adding the 100μl of preblocked antibody-bead complexes per sample and incubating overnight at 4°C on a rotator. Elution and crosslink reversal of both sample and input was performed by incubating the samples for 6 hours at 65°C in a waterbath. The DNA was purified using the Zymo ChIP DNA Clean & Concentrator kit (Zymo Research) according to the manufacturer’s instructions. The sequencing library was generated using the MicroPlex Library Preparation kit (Diagenode) and size selected using Agencourt AMPure XP. The libraries were quantified using the qPCR based Illumina library quantification kit (Kappa Biosystems) and 75bp single end sequenced to a high depth (~50M reads) on a NextSeq500 platform (Illumina). Reads were aligned to the human genome (GRCh37 via Ensembl release 72) using BWA. Peaks were identified using MACS v1.4.2. Downstream processing was performed in R using the Bioconductor packages DiffBind, ChIPQC and Gviz. Gene Ontology terms and associations were taken from Bioconductor annotation packages Go.db and org.HS.eg.db. Motif enrichment was performed using MEME. ChIPseq raw data are available in the ArrayExpress database under accession number E-MTAB-3137.

## Results

### A mesenchymal-epithelial transition in RPE

**Fig 1:**
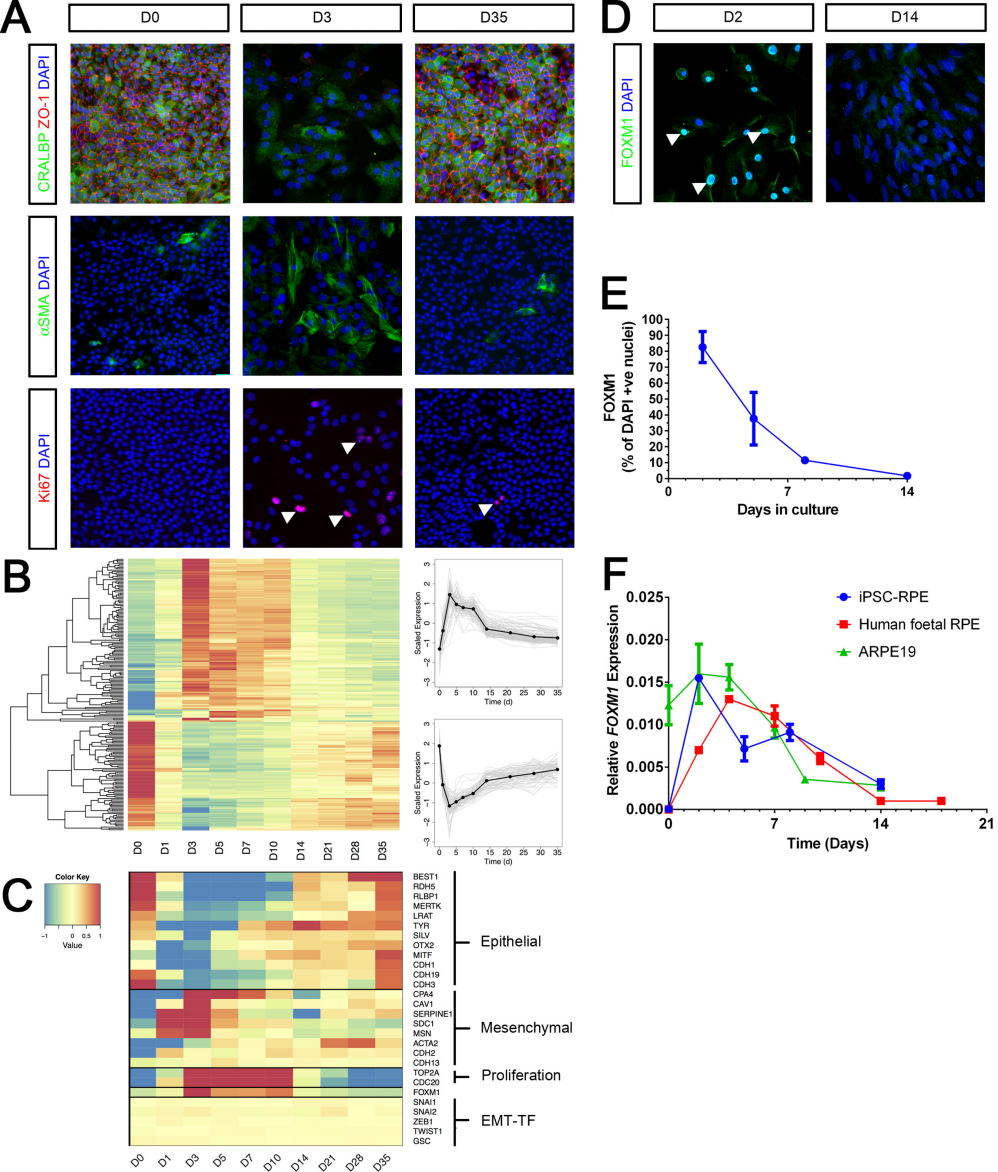
A Mesenchymal-Epithelial Transition with temporal FOXM1 expression during RPE culture. A. Immunocytochemistry was performed for CRALBP, ZO1, αSMA and Ki67 at Day 0 (D0), Day 3 (D3) and Day 35 (D35). Arrowheads point towards Ki67 positive nuclei. B. Microarray heatmap of the expression profiles of the top 250 genes, ranked by the significance of their expression changes, over time in culture. Raw expression data are mean centred and scaled to unit variance prior to clustering. A schematic of the scaled expression is shown on the right where individual gene profiles are in light grey and the mean expression profile is shown in black. C. Microarray heatmap showing transcript expression for a panel of representative markers over a timecourse of RPE culture. D. Immunocytochemistry for FOXM1 at Day 2 and Day 14 of RPE culture. Arrowheads point towards FOXM1 positive nuclei. E. Quantification of immunocytochemistry showing percentage of nuclei staining positive for FOXM1 over time. Bars represent Mean ± SD (n = 3). F. Expression of *FOXM1* transcript measured using qPCR (relative to housekeeping genes *ACTB* and *GAPDH*) in iPSC derived RPE, human foetal RPE and ARPE19 cells over time. Bars represent Mean ± SD (n = 3)

RPE derived from pluripotent stem cells display a characteristic cobblestone-shaped morphology visualized by immunostaining for ZO-1, a marker of cell junctions in epithelial cells and express proteins associated with RPE function such as the cellular retinaldehyde-binding protein (CRALBP) (Fig 1A, left). During *in-vitro* culture, where cells are dissociated to break intercellular contacts, RPE proliferate and acquire a de-differentiated, mesenchymal-like morphology expressing high levels of the proliferation marker Ki67 and alpha-smooth muscle actin (α-SMA) (Fig 1A, middle). This mesenchymal state then transitions into an epithelial state through a Mesenchymal-Epithelial Transition (MET), resulting in re-expression of epithelial markers (Fig 1A, right). In order to gain further understanding of this transition, we profiled the global gene expression on Days 1,3,7,10,14,21,28 and 35 of culture which captured different stages of the MET process. A striking feature of the transcriptional data was the clustering of genes into two distinct, symmetrical clusters as can be seen by hierarchical clustering of the top 250 expressed genes (Fig 1B). The first cluster consists of genes that are highly expressed in RPE before culture (D0) but show an immediate drop in expression followed by a steady increase back towards the starting level whereas the second cluster shows the inverse pattern. This shows that RPE culture is associated with dynamic gene expression and cellular state changes. We then looked specifically at the expression of markers representative of the epithelial and mesenchymal phenotype. There was an upregulation of mesenchymal markers such as *CDH2*, *SERPINE1*, *SDC1*, *MSN* concomitant with a decrease in epithelial markers such as *CDH1*, *CDH3*, *OTX2*, *MITF* and *TYR*, even at the earliest timepoint profiled, compared to the starting RPE population prior to dissociation and culture (Fig 1C). This shows that there was a very rapid uptake of mesenchymal characteristics in response to cellular dissociation. However, the mesenchymal-epithelial transition was more gradual with gene expression returning to the pre-culture transcriptional state over the following 2–5 weeks. These expression profiles were confirmed by gene-specific qPCR for representative genes (S1A Fig). The initial stage of this MET also overlapped with a period of high proliferation as seen by increased expression of markers such as *TOP2A* and *CDC20* (Fig 1C). This was further supported by gene ontology analysis which illustrated enrichment of gene sets involved in proliferation (cell cycle, DNA replication and ribosome biogenesis) alongside an under-representation of those associated with RPE function (melanin biosynthesis and visual perception) during early stage MET (S1B Fig).

MET is usually associated with a downregulation of classical EMT-TFs such as Snail (SNAI1), Slug (*S*NAI2), ZEB1, TWIST1 and GSC that are upregulated during an epithelial-mesenchymal state change. Remarkably, there was no change in expression of these factors following dissociation and culture of RPE (Fig 1C) even though phenotypic and molecular characteristics of a MET were fulfilled. In contrast, we noted that the expression pattern of the forkhead transcription factor FOXM1 dynamically changed during culture ranging from elevated expression during initial stages of the MET to low expression in epithelial RPE (Fig 1C). We validated this expression pattern by immunostaining which confirmed that FOXM1 was highly expressed within the first week of culture and had a nuclear localization, in line with its predicted role as a transcription factor (Fig 1D and 1E). We found an identical FOXM1 expression profile in RPE obtained from other sources e.g RPE derived from iPS cells, human foetal RPE and the cell line ARPE19, suggesting that temporal FOXM1 expression is a common feature of RPE cells (Fig 1F).

### FOXM1 regulates epithelial fate acquisition of RPE

**Fig 2:**
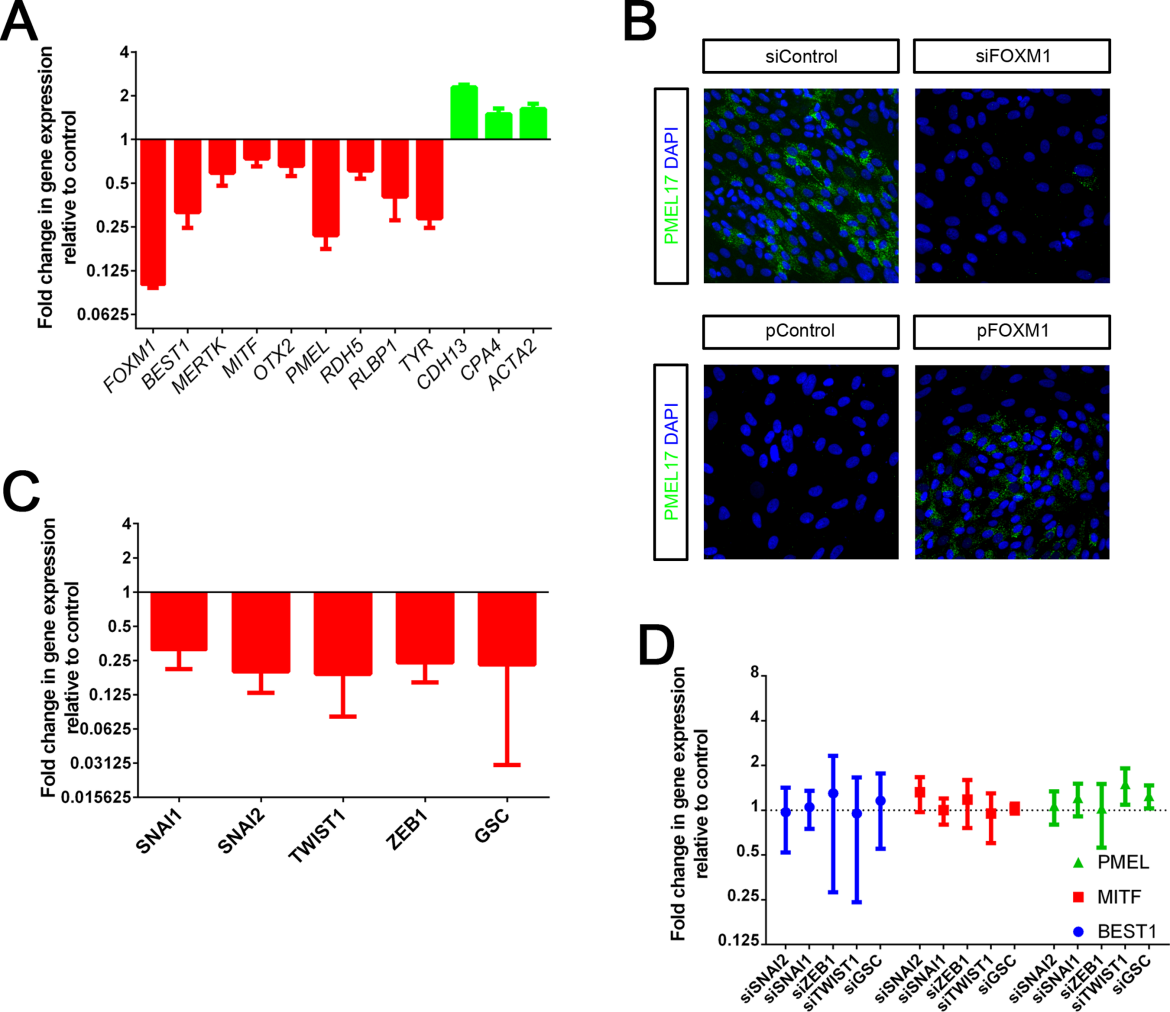
FOXM1 promotes RPE epithelial fate. A. qPCR based measurement of transcript expression of a panel of epithelial (red) and mesenchymal (green) markers at Day 10 post siFOXM1 transfection (except levels of *FOXM1* itself which are measured at Day 2 post knockdown). Data is normalized to transfection with non-targeting siRNA used as a control. *ACTB*, *GAPDH*, *IPO8* and *HPRT1* are used as housekeeping genes. Bars represent Mean + SD (n = 3). P<0.05 (Student’s t-test). B. Immunocytochemistry for PMEL17 upon FOXM1 knockdown (siFOXM1) or overexpression (pFOXM1) at Day 10 post transfection. C. Level of knockdown obtained upon transient transfection of siRNA against SNAI2, SNAI1, ZEB1, TWIST1 and GSC. Knockdown was measured by qPCR at Day 6 post transfection and is expressed relative to non-targeting siRNA used as control. *CYC1* and *GAPDH* were used as housekeeping genes. Bars represent Mean ± SD (n = 6–9). Knockdown of EMT-TF expression was significant, P<0.05 (Student’s t-test). D. No significant effect on *PMEL*, *MITF* or *BEST1* expression was observed under the same conditions described above for Fig 2C.

In order to examine the role of FOXM1, we asked whether modulation of FOXM1 levels affected eventual acquisition of the epithelial phenotype. To address this, we performed transient knockdown of FOXM1 and assessed the effect on expression of epithelial and mesenchymal markers. We observed a decrease in epithelial and an increase in mesenchymal marker expression upon FOXM1 knockdown at Day 10 post-transfection (Fig 2A and 2B); a stage where normally cells start to re-express epithelial and downregulate mesenchymal markers. This suggests that loss of FOXM1 renders cells deficient in their capability to undergo a successful MET leading to a failure in achieving an epithelial phenotype. Conversely, overexpression of FOXM1 resulted in an increase in epithelial phenotype as seen by increased expression of the premelanosomal protein PMEL17, a marker of pigmented RPE (Fig 2B). These effects were not seen upon knockdown of SNAI1, SNAI2, TWIST1, ZEB1 or GSC which were unaltered in expression during RPE culture (Fig 2C and 2D). This confirmed that constancy of transcript levels of these classical EMT-TFs also translated to no observable functional requirement. Taken together, these data highlight that FOXM1 expression, even though restricted to a transient period of RPE culture, has an important effect on transition from the mesenchymal to the epithelial state and is required for successful epithelial fate acquisition in RPE.

### FOXM1 regulates RPE proliferation

**Fig 3:**
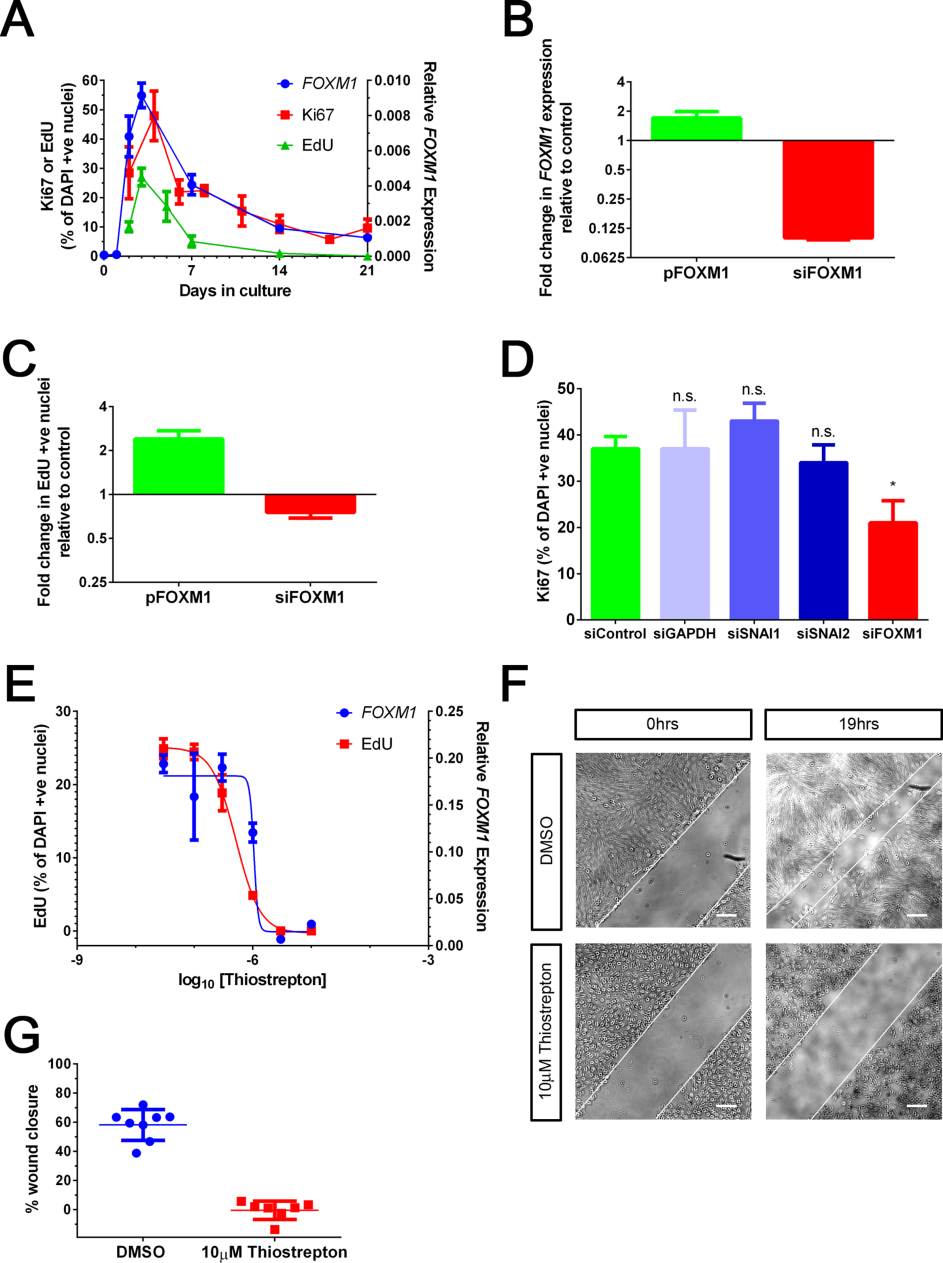
FOXM1 regulates RPE proliferation. A. Graph showing quantification of immunocytochemistry where % Ki67 (n = 3) or % EdU (n = 6) is plotted on the left Y axis and relative expression of *FOXM1* transcript (n = 3; *ACTB* used as housekeeping gene) on the right Y axis over days in culture (x axis). B. Quantification of change in *FOXM1* transcript upon transient overexpression (pFOXM1) or knockdown (siFOXM1), 48h post transfection, measured by qPCR. Data is normalized to appropriate controls (Empty vector for pFOXM1 and non-targeting siRNA for siFOXM1). Bars represent Mean + SD (n = 3). C. Quantification of change in EdU incorporation upon FOXM1 overexpression or knockdown, 72h post transfection. Data is normalized to appropriate controls (Empty vector for pFOXM1 and non-targeting siRNA for siFOXM1). Bars represent Mean + SD (n = 4). P<0.0001 (Student’s t-test). D. Quantification of immunocytochemistry for Ki67 upon siRNA mediated knockdown of non-targeting control, GAPDH, SNAI1, SNAI2 and FOXM1, at Day 6 post transfection. Bars represent Mean + SD (n = 3). n.s non-significant, * p<0.05 Student’s t-test. E. Effect of Thiostrepton on EdU incorporation [left Y axis, red] and *FOXM1* transcript expression measured by qPCR [right Y axis, blue]. Bars represent Mean ± SD (n = 6). F. Bright-field microscopy showing a scratch introduced in a RPE monolayer at 0 hrs and 19hrs in the presence of DMSO or 10μM Thiostrepton. Edge of the scratch is marked with a white line. Scale bar = 200 μm. G. Quantification of F (above). Bars represent Mean + SD (n = 7). P<0.0001 (Student’s t-test)

With a view to gain further insight into the role of FOXM1, we followed up on our initial observation that temporal expression of FOXM1 overlapped with a period of high proliferation (Fig 1C). This was further verified by immunostaining for Ki67 as well as through incorporation of EdU, a modified thymidine analogue, into DNA (Fig 3A). Therefore, we investigated whether FOXM1 regulates proliferation during the initial phase of MET. We modulated the expression of FOXM1 either by transient knockdown or overexpression (Fig 3B) and quantified the effect on proliferation using EdU incorporation as the output measure. An increase in proliferation was observed when FOXM1 was overexpressed whereas the proliferation decreased upon FOXM1 knockdown (Fig 3C). No change in proliferation was observed by knockdown of SNAI1 or SNAI2 reiterating no role of these factors in this process (Fig 3D). To further interrogate the role of FOXM1, we used the thiazole antibiotic Thiostrepton which has been shown to inhibit FOXM1 expression as well as its DNA-binding capacity [33]. RPE cells were treated with a range of Thiostrepton concentrations for a period of 48 hours followed by measurement of FOXM1 transcript expression and the level of cell proliferation. Thiostrepton treatment resulted in a reduction in FOXM1 expression which correlated with a decrease in EdU incorporation with comparable IC_50_ values (1.1 μM and 0.6 μM respectively) (Fig 3E). This indicates that the inhibitor has a similar efficacy for both readouts and is consistent with its IC_50_ estimated in previous studies [34,35]. In addition, we performed an *in-vitro* wound closure assay as an alternative way to measure proliferation and migration potential of epithelial cells. A scratch wound was introduced in a confluent RPE monolayer and cells were treated with either vehicle or Thiostrepton. The closure of the wound was measured after a period of 24 hours. As expected, Thiostrepton treated cells were deficient in their ability to close the wound suggesting a reduction in their proliferative and migratory capacity (Fig 3F and 3G). Taken together, our data show that both biological and chemical manipulation of FOXM1 function affects the proliferative capacity of RPE, consistent with the hypothesis that FOXM1 is a key regulator of RPE proliferation.

### FOXM1 directly targets proliferation associated genes

**Fig 4:**
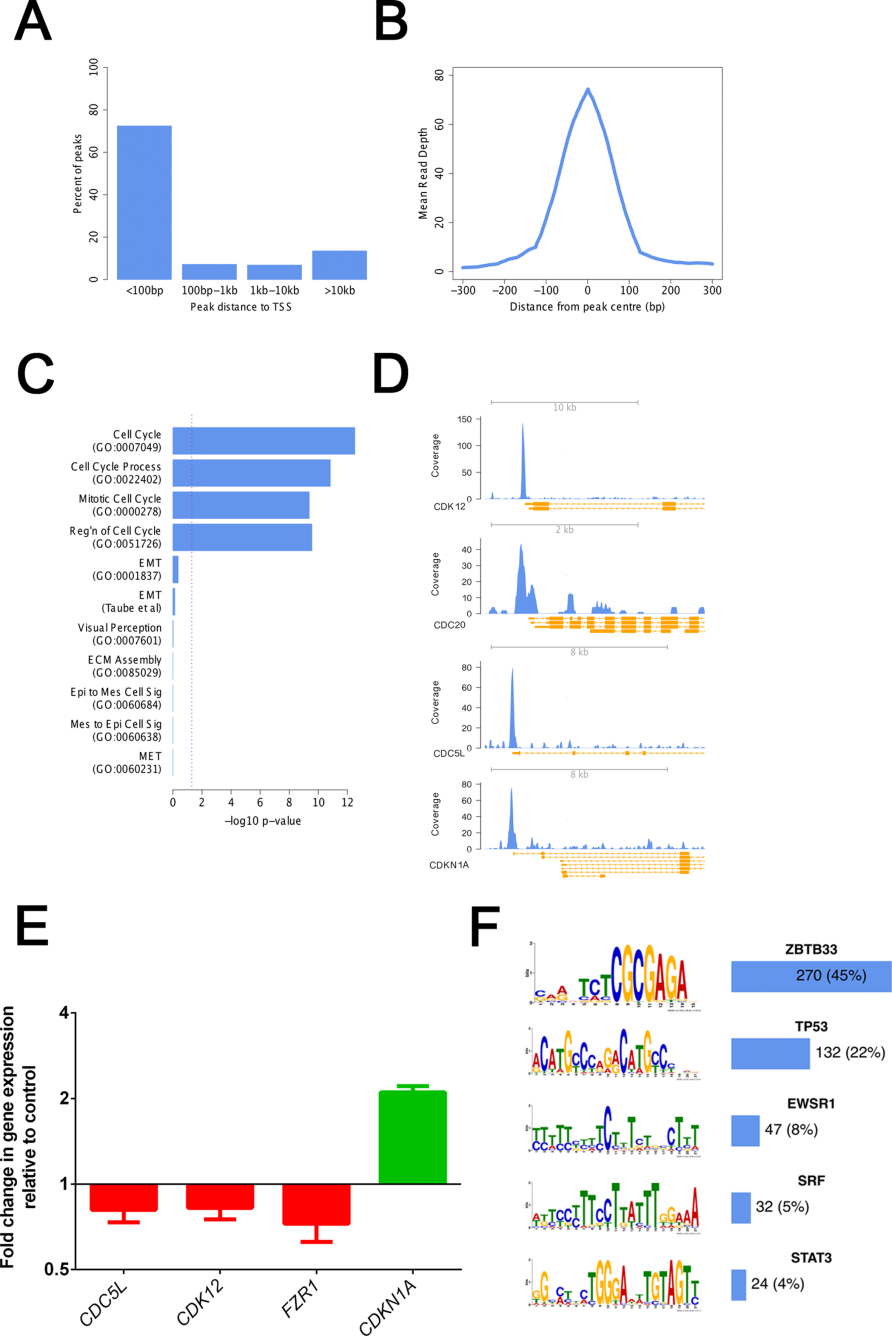
FOXM1 binds to promoters of proliferation associated genes. A. Percentage of FOXM1 peaks within proximity boundaries to Transcription Start Sites (TSS). B. Plot showing the mean read depth over FOXM1 peaks with a majority of binding within 100bp of peak centres. C. Protein coding genes with a FOXM1 peak within 1kb of TSS are highly enriched for GO categories relevant to cell cycle related functions but not EMT, MET, epithelial or mesenchymal related functions. In addition to the GO category, enrichment was also tested at a published EMT gene signature [55] with no significant binding seen. Enrichment was calculated using a hypergeometric distribution, the—log_10_ p-value is shown. Dashed line represents p = 0.05. D. Schematic showing FOXM1 binding to the promoters of representative cell cycle genes; CDK12, CDC20, CDC5L & CDKN1A. ChIP-seq coverage is shown in blue and annotated genomic features shown in orange. E. Quantification of change in transcript expression of representative FOXM1 bound genes, measured by qPCR, upon siRNA mediated FOXM1 knockdown (relative to transfection with non-targeting siRNA used as a control), 72h post transfection. *ACTB* is used as a housekeeping gene. Bars represent Mean + SD (n = 3). P<0.05 (Student’s t-test). F. Significantly enriched transcription factor motifs in FOXM1 peaks alongside frequencies of occurrence.

In order to gain a mechanistic understanding of how FOXM1 regulates RPE proliferation, MET and epithelial fate acquisition, we performed chromatin immunoprecipitation (ChIP) followed by deep sequencing to examine its genome-wide binding pattern in an unbiased manner. For robust identification, we selected peaks which were identified in two independent experiments and at least 500 bp away from any false positive found using non-specific IgG. Using these parameters, 599 statistically significant and reproducible FOXM1 binding sites were identified. Of these, 434 (73%) were within 100 bp and 477 (80%) within 1kb of transcriptional start sites (TSS) of protein coding genes (Fig 4A) with a tall and narrow binding pattern (Fig 4B) suggesting a direct regulatory role of FOXM1 in gene transcription. The expression profile of these genes during the MET timecourse showed high expression during the early timepoints and a decrease in expression with time in culture on average, which resembled the profile seen for FOXM1 transcript and protein (S2 Fig). GO functional analysis of FOXM1 bound genes revealed enrichment of categories relevant to proliferation (Fig 4C). However, no significant binding was found at genes involved in MET, EMT or their respective signalling pathways. There was also no binding to genes regulating the RPE/epithelial or mesenchymal phenotype e.g CDH1 or CDH2. The link to proliferation was further supported by association of FOXM1 with promoters of genes known to regulate cell cycle progression e.g CDK12, CDC20, CDKN1A, CDC5L (Fig 4D). siRNA mediated knockdown of FOXM1 resulted in decreased expression of positive regulators of the cell cycle (CDC5L, CDK12 and FZR1) and an increase in expression of CDKN1A, a known inhibitor of the cell cycle (Fig 4E). This indicates that FOXM1 promotes proliferation by either acting as an activator or repressor in a gene dependent manner, consistent with previous reports [28,36,37].

To ascertain whether there were unique features associated with FOXM1 binding sites, we performed motif enrichment analysis to look for known or novel motifs. We identified 5 motifs that were enriched in FOXM1 bound sequences which are also known motifs for other transcription factors; ZBTB33, TP53, EWSR1, SRF and STAT3 (Fig 4F). A binding interaction between ZBTB33 (Kaiso) and FOXM1 has been identified previously [38] which might explain the high incidence of this motif within our peakset and point towards a co-regulatory role of these proteins.

From these data, we concluded that the major role of FOXM1 is to regulate proliferation via genes affecting cell cycle progression; and not in directly regulating genes involved in mesenchymal to epithelial transition or phenotypic determination of the mesenchymal and epithelial state.

### Role of the BMP/Wnt signalling axis in MET

**Fig 5:**
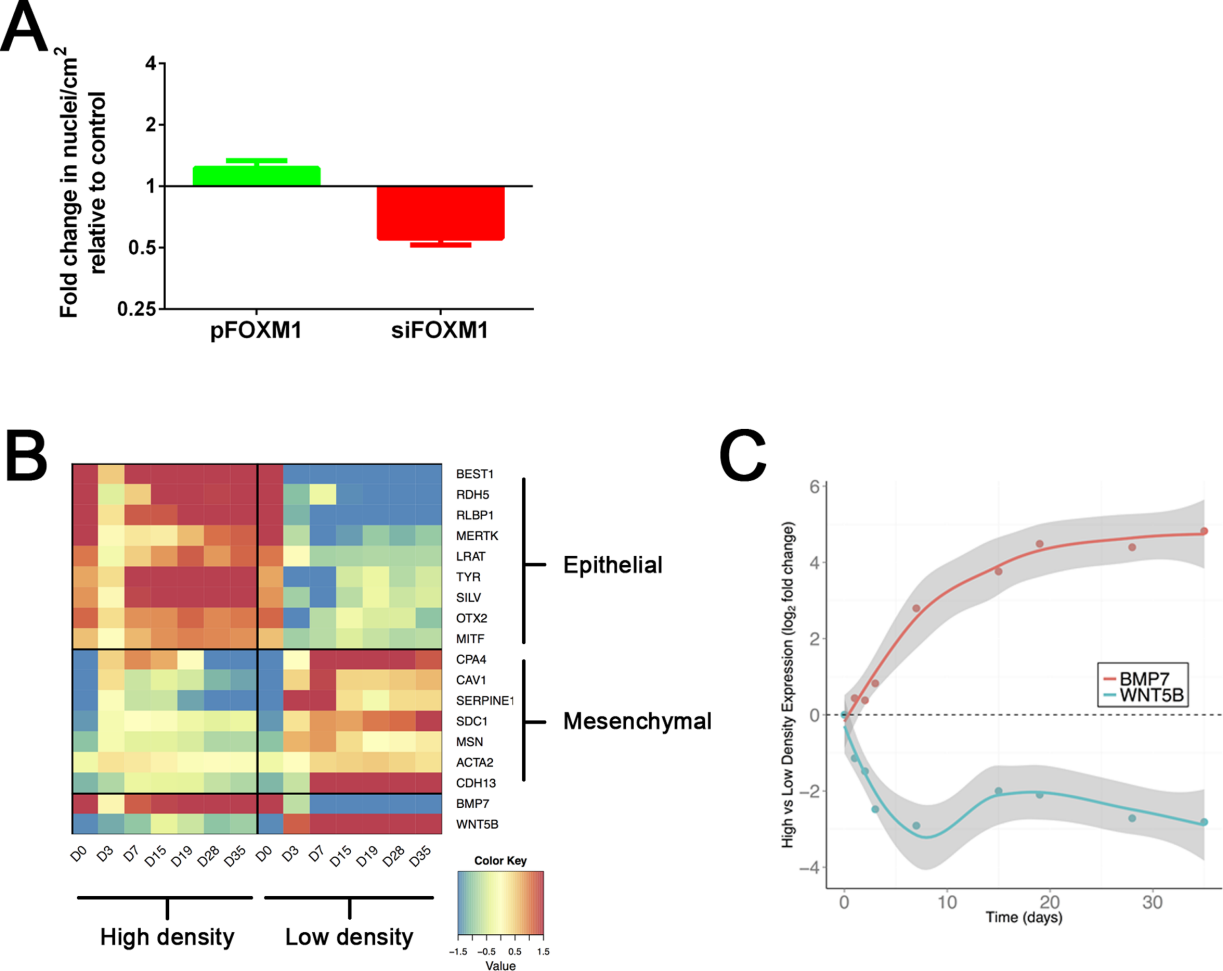
Epithelial fate acquisition is density dependent. A. Quantification of change in cell density (number of DAPI positive nuclei per cm^2^ imaged area) upon FOXM1 overexpression or knockdown, 72h post transfection. Data is normalized to appropriate controls (Empty vector for pFOXM1 and non-targeting siRNA for siFOXM1). Bars represent Mean + SD (n = 4). P<0.0001 (Student’s t-test). B. Heatmap showing changes in gene expression of a panel of representative markers over a timecourse of RPE culture where cells are seeded at high (100000 cells/cm^2^) or low (8000 cells/cm^2^) density. C. Plot showing differential expression of *BMP7* and *Wnt5B* transcripts extrapolated from the microarray data. The shaded area represents 95% confidence intervals around the point estimates (circles) of the difference between the mean high density expression vs the mean low density expression.

Following on from our observation that FOXM1 was required for a successful MET and that its major function was regulation of proliferation, we wanted to understand the relationship between proliferation and epithelialization in greater detail. Therefore, we asked the question: how does regulation of proliferation translate into regulation of the epithelial state? To address this, we first explored the observation that modulating FOXM1 levels, besides affecting proliferation, also resulted in a change in cell density. This was evidenced by higher or lower number of Hoechst positive nuclei per cm^2^ of surface area upon FOXM1 overexpression or knockdown respectively (Fig 5A). Additionally, we observed that acquisition of the epithelial phenotype was critically reliant on the cell plating density, where cells seeded at high density regained epithelial markers after transiting through a MET whereas cells seeded at low density remained mesenchymal-like. This was confirmed by microarray profiling across different timepoints of RPE cultures seeded at either high (100000 cells/cm^2^) or low (8000 cells/cm^2^) density which showed that low density cultures continued to remain in a mesenchymal, de-differentiated state at the end of culture (Fig 5B). This was also verified by gene specific qPCR for representative markers which confirmed that low density cultures expressed high levels of mesenchymal markers and low levels of epithelial markers compared to cultures seeded at high density (S3 Fig). Therefore, affecting proliferation had an effect on cell density which in turn is a crucial factor for epithelial fate acquisition. A major difference between a high and low density culture is that cells are in closer spatial proximity in the former which promotes formation of adhesive interactions which subsequently promote epithelial acquisition. Furthermore, cells secrete soluble signals that can alter the microenvironment and strongly affect epithelial homeostasis. Therefore, we conjectured that there must be signalling factors that are differentially expressed between high and low density cultures that enable cells to communicate their spatial context and allow epithelial gene expression to occur in a coordinated manner. To investigate this hypothesis further, we interrogated the transcriptome timecourse to look for signalling ligands that were at least 2 fold differentially expressed between the high and low density cultures at day 35 with a False Discovery Rate (FDR) < 0.01. We found 122 genes that met this criteria (S1 Table) from which we focussed on BMP7 and the non-canonical Wnt ligand Wnt5B for further exploration. BMP7 and Wnt5B are expressed in the high and low density cultures with a reciprocal expression pattern such that low density cultures express high levels of Wnt5B and low levels of BMP7(Fig 5B bottom and 5C). This expression profile was confirmed by gene specific qPCR (S4 Fig). Furthermore, we performed causal reasoning analysis of all genes differentially expressed at day 35 (FDR < 0.01, >2 fold change), which predicts the regulators responsible for the observed gene expression profiles [39]. This analysis showed an enrichment of genes downstream of BMP7 signalling (hypergeometric p < 1e-9) and Wnt5B signalling (hypergeometric p < 0.001) indicating that the downstream pathway was appropriately altered. Taken together, this suggests that successful MET requires high BMP7 expression and signalling concomitant with low Wnt5B expression and its downstream signalling.

**Fig 6:**
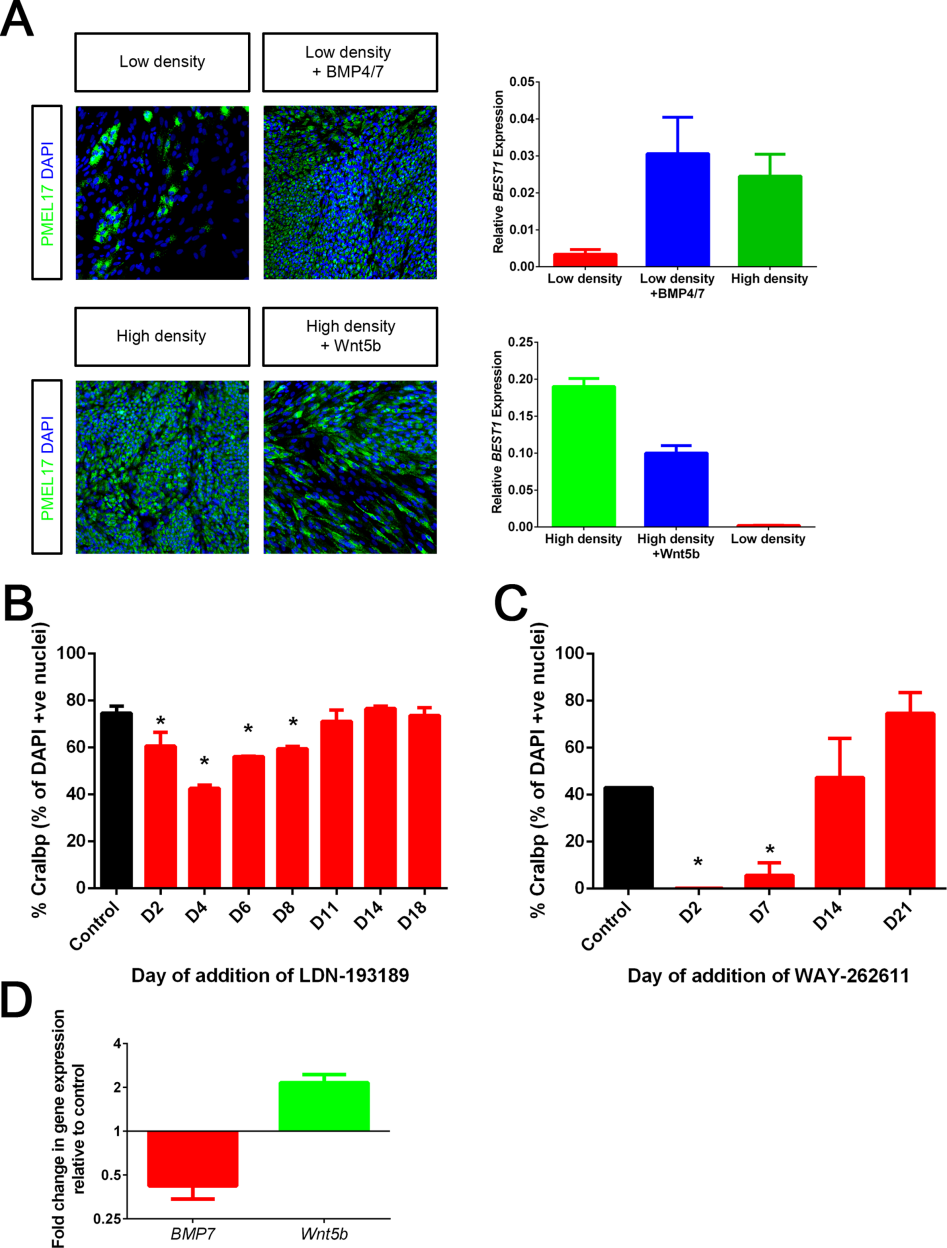
BMP/Wnt signalling is required for MET. A. Immunocytochemistry for PMEL17 where cells are seeded at either low density (16000 cells/cm^2^) in the presence or absence of BMP4/7 (top left) or at high density (25000 cells/cm^2^) in the presence or absence of Wnt5B (bottom left) and cultured for a period of 14 days. Also shown is the expression of *BEST1* under the same conditions (top and bottom right). *ACTB* and *B2M* are used as housekeeping genes. Bars represent Mean + SD (n = 3). B. Quantification of immunocytochemistry for % CRALBP at Day 21 where cells are either treated with media alone (Control) or media supplemented with 10μM LDN-193189 added at Day 2,4,6,8,11,14 or 18. * indicates significant difference between control and compound treatment (One way ANOVA with Dunnett’s multiple comparisons). C. Quantification of immunocytochemistry for % CRALBP at Day 28 where cells are either treated with media alone (Control) or media supplemented with 10μM WAY-262611 added at Day 2,7,14 or 21. * indicates significant difference between control and compound treatment (One way ANOVA with Dunnett’s multiple comparisons). D. qPCR based measurement of *BMP7* and *Wnt5B* transcript expression at Day 10 post siFOXM1 transfection (relative to transfection with non-targeting siRNA used as a control). *GAPDH*, *HPRT1* and *IPO8* were used as housekeeping genes. Bars represent Mean + SD (n = 3). P<0.05 (Student’s t-test).

Following on, we asked whether BMP7 and Wnt5B were *bona-fide* signalling effectors in this culture system i.e were they capable of signalling and affecting epithelial fate when added exogenously? Indeed, addition of recombinant BMP4/7 heterodimer, which is more potent at inducing BMP7 signalling than BMPs added in isolation or in their homodimeric form [40], rescued the non-epithelial phenotype of low density cultures by increasing expression of the RPE markers PMEL17 and BEST1 (Bestrophin) to levels similar to those of high density cultures (Fig 6A, top). Similarly, exogenous addition of recombinant Wnt5B reduced expression of epithelial markers making the cells less epithelial-like (Fig 6A, bottom). These data suggested that BMP7 and Wnt5B were capable of extracellular signalling and affecting epithelial fate in an antagonistic manner. Next, we investigated the stage of RPE culture where these pathways played a role by using small molecules to modulate endogenous signalling. LDN-193189, an inhibitor of BMP signalling [41] was added to RPE at different stages of culture and maintained in the media until Day 21. The effect on epithelial phenotype was measured by immunostaining for CRALBP. We observed a reduction in CRALBP expression when LDN-193189 was introduced at any stage between Day 2 and Day 11 (i.e added to the media at D2, D4, D6 or D8), which was not seen at other timepoints (Fig 6B). As shown before, this stage is associated with proliferation and mesenchymal-epithelial gene expression transitions. Therefore, this substantiated the hypothesis that active BMP signalling is required during the initial stages of RPE culture for successful MET and epithelial fate acquisition. Similarly, we introduced WAY-262611, a DKK1 inhibitor which activates Wnt signalling [42], at different stages of culture and observed that Wnt activation between Day 2 and Day 14 (i.e compound addition at D2 or D7) led to a reduction in CRALBP expression (Fig 6C). This supports the hypothesis that Wnt signalling, in contrast to BMP signalling, is suppressed during early culture to enable reuptake of epithelial phenotype. Off-target effects due to compound toxicity were ruled out because compound addition at later timepoints did not have detrimental effects on CRALBP expression. Taken together, this data supports the notion that BMP and WNT signalling act in a reciprocal and antagonistic manner to promote MET and achievement of the epithelial fate.

Finally, we investigated whether FOXM1 had any effect on the BMP/Wnt signalling axis. Knockdown of FOXM1 resulted in a decrease in expression of BMP7 and an increase in expression of Wnt5B (Fig 6D). This indicates that FOXM1 positively affects BMP signalling and negatively affects Wnt signalling and may play a role in balancing these opposing cues.

In summary, our study comprehensively characterizes the mesenchymal-epithelial transition that occurs in RPE culture. We show that this transition is not regulated through canonical EMT-TFs such as Snail and Slug and instead can be explained through FOXM1 dependent control of proliferation and as well as its effect on the BMP/Wnt signalling axis.

## Discussion

The RPE monolayer, formed in the early embryo, is a terminally differentiated cell sheet which normally remains non-proliferative throughout life. However, in certain ocular diseases such as Proliferative Vitreoretinopathy (PVR) and during *in-vitro* culture, their quiescence can be released resulting in a re-entry into the cell-cycle and proliferation. This has detrimental effects in the context of pathologies such as PVR, as it leads to abnormal proliferation resulting in the formation of fibrotic scar tissue and contraction of the retina thereby compromising vision. However, this very feature can be harnessed for therapeutic use as it allows scale-up and expansion of cells in controlled laboratory conditions.

hESC-derived RPE are currently being used for transplantation in clinical trials for macular dystrophy with promising initial results making them a very topical and relevant cell type for further investigation. However, these therapeutic strategies rely on the proliferative capacity of RPE to generate sufficient material for transplantation. Furthermore, introduction of a single-cell RPE suspension to replace diseased cells, which is one of the approaches currently being clinically tested [43,44], potentially involves proliferation of RPE upon transplantation into the patient *in-vivo*. Therefore, it is crucial to gain detailed mechanistic understanding of how this proliferation is regulated and how it relates to re-acquisition of the epithelial state in order to better understand clinical outcome.

In this study, we have extensively characterized the transitions that occur when hESC-derived RPE are cultured. We show that dissociated RPE deviate from an epithelial state and instead express hallmarks of mesenchymal cells during early culture periods. The uptake of mesenchymal characteristics is very rapid and potentially triggered due to breaking of cell-cell contacts. The molecular regulators of this early epithelial to mesenchymal transition are currently not identified and would be an important avenue for further study. Once dissociated and cultured, RPE transition from the mesenchymal state to re-establish an epithelial phenotype in a density-dependent manner. This mesenchymal-epithelial transition is accompanied by molecular changes that are characteristic of such transitions e.g switch in expression of N-cadherin (CDH2) to epithelial/RPE specific cadherins such as CDH1 and CDH3. Although an EMT-MET like change has been reported in some RPE studies previously [12,45,46], this is, to our knowledge, the first comprehensive study covering the entire spectrum of transitional states during RPE culture. Furthermore, transcriptomic studies of this process have been limited to cell lines such as ARPE19 [47,48] which have debatable relevance to native RPE.

**Fig 7:**
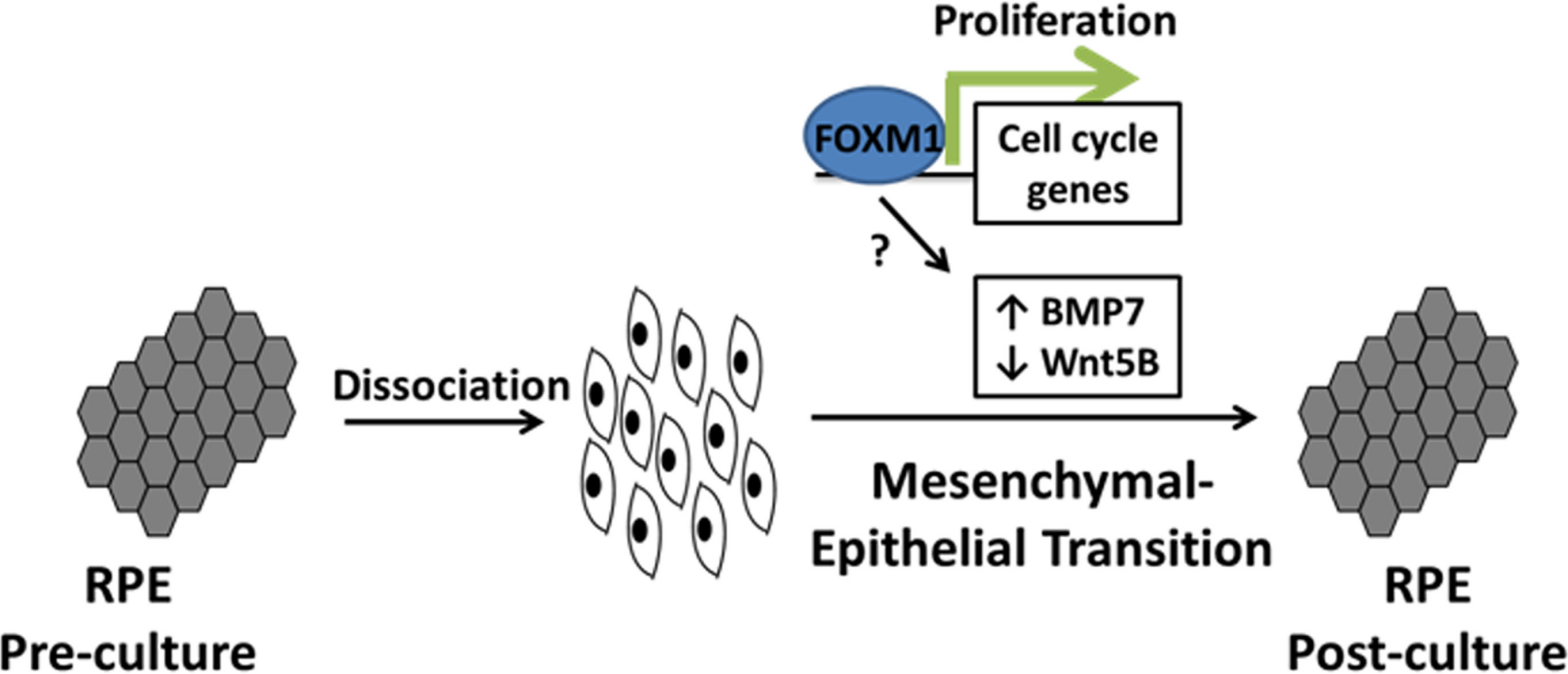
Model showing proposed roles of FOXM1 in epithelial fate acquisition. RPE first acquire a mesenchymal morphology upon dissociation and culture followed by proliferation and mesenchymal-epithelial transition to re-uptake an epithelial phenotype. Proliferation of RPE is directly regulated by FOXM1 which also affects expression of BMP7 and Wnt5B by an unknown mechanism. Both these activities are required for successful MET and epithelialization.

Our results demonstrate for the first time that the proto-oncogene FOXM1 plays an important role in epithelial fate determination of RPE. As shown in Fig 7, we propose that this is achieved by two modes; first by directly regulating proliferation of RPE facilitated through direct binding at cell cycle gene promoters. Proliferation and resultant spatial context of cells is required for an efficient epithelial gene expression program to be initiated. Secondly, FOXM1 modulates expression of signalling factors BMP7 and Wnt5B whose mutually antagonistic signalling profile is required for successful mesenchymal-epithelial transition. The reciprocal crosstalk between the BMP/Wnt pathway and its link to RPE MET resonates with a similar requirement of this signalling axis in self-renewal and MET in other cellular systems [49,50].

We are cognizant that our data does not support FOXM1 binding directly to promoters of genes that govern the epithelial phenotype and hence their direct transcriptional control. Instead, we show data that support the hypothesis that effect on epithelial fate acquisition is a consequence of its effect on proliferation. Further experiments are warranted to better understand the molecular sequelae underpinning the MET. For example, it will be important to determine whether there are other regulators that affect the epithelial transcriptional program more directly. Similarly, the effect of FOXM1 on BMP7 and Wnt5B may also be indirect as no binding was detected at their promoters. This may occur either through affecting other upstream factors or post-transcriptionally as has been described for FOXM1’s regulation of the SMAD3/4 complex [51]. In this regard, it is noteworthy that our analysis of FOXM1 bound promoter sequences highlighted the enrichment of the motif for ZBTB33, a known repressor of non-canonical Wnt signalling [52], also found to interact physically with FOXM1 in a previous report [38]. This suggests that FOXM1 and ZBTB33 might exist in a complex and this interaction may mediate co-repression of genes such as Wnt5B. It is possible that in some instances e.g at the promoter of Wnt5B, DNA binding is mediated through ZBTB33 or other FOXM1 interacting proteins rather than FOXM1 itself. Such indirect interactions may be transient and would not be captured in a typical ChIP Seq experiment which may explain the absence of FOXM1 peaks at the promoter. During the course of our study, we were intrigued to note the temporal nature of FOXM1 expression which is tightly regulated and coupled to proliferation. Further investigation into the role of FOXM1 using RPE as a model system may facilitate identification of signalling pathways and factors that control its precise expression in a healthy cell. This would be of immense interest in the field of oncology as FOXM1 is frequently overexpressed in several cancers [53]. We also provide a valuable dataset in terms of identifying FOXM1 binding sites in a healthy genome. This contrasts with previously published ChIP-Seq datasets using cancer cell lines that represent perturbed, transformed systems where the majority of FOXM1 binding is detected at intergenic and intronic regions [32,54]. Further study of FOXM1 may also be relevant to ocular pathologies such as PVR where normally quiescent RPE undergo abnormal proliferation resulting in the formation of fibrotic scar tissue and contraction of the retina thereby compromising vision. Our data also lends support to the notion that successful therapeutic use of stem cell derived RPE may require implantation of a cell sheet rather than cells in suspension which have the capacity to proliferate and undertake a de-differentiated, non- functional state.

To summarize, we describe the control of epithelial proliferation by FOXM1 and requirement of coordinated interplay of signalling factors to achieve expression of epithelial phenotype. We show that FOXM1 is required for a successful Mesenchymal-Epithelial Transition in RPE in contrast to any role of classical EMT inducing transcription factors. We also present the use of stem cell derived RPE as a previously under-utilized resource to gain insight into mechanisms that may underpin common themes in diverse processes such cancer and metastasis.

## Supporting Information

S1 Fig Gene expression in RPE culture. A. qPCR quantification of transcript expression (relative to Day 0) of epithelial and mesenchymal markers over a timecourse of RPE culture. *ATP5B* and *CYC1* are used as housekeeping genes. Bars represent Mean ± SD (n = 3). B. Gene set test significance P values for exemplar GO terms for Day 3 versus Day 0 (P < 0.05 for all terms). High values indicate up-regulation at Day 3 relative to Day 0 and low values indicate down-regulation.
(TIF)

S2 Fig Mean expression profile of FOXM1-bound genes in RPE dissociated and cultured for a period of 35 days. The raw microarray expression data of all genes containing FOXM1 peaks was mean centered and scaled to unit variance prior to plotting. Bars represent standard error.
(TIF)

S3 Fig Expression of epithelial and mesenchymal genes in a density dependent manner RPE culture. qPCR quantification of transcript expression (relative to Day 0) of epithelial (top) and mesenchymal markers (bottom) over a timecourse of RPE culture where cells are seeded either at high (100000 cells/cm^2^) or low (8000 cells/cm^2^) density. *ATP5B* and *CYC1* are used as housekeeping genes. Bars represent Mean ± SD (n = 3).
(TIF)

S4 Fig BMP7 and Wnt5B signalling in RPE. qPCR quantification of transcript expression of BMP7 (top) and Wnt5B (bottom) over a timecourse of RPE culture where cells are seeded either at high (100000 cells/cm^2^) or low (8000 cells/cm^2^) density. *ACTB* is used as a housekeeping gene. Bars represent Mean ± SD (n = 3).
(TIF)

S1 Table Table containing list of genes differentially expressed between high and low density cultures at Day35. (XLSX)
