# Supplementary figures and images for: A FOXM1 Dependent Mesenchymal-Epithelial Transition in Retinal Pigment Epithelium Cells
Source: PLoS One. 2015 Jun 29;10(6):e0130379. doi: 10.1371/journal.pone.0130379 (PMC4488273; doi:10.1371/journal.pone.0130379)

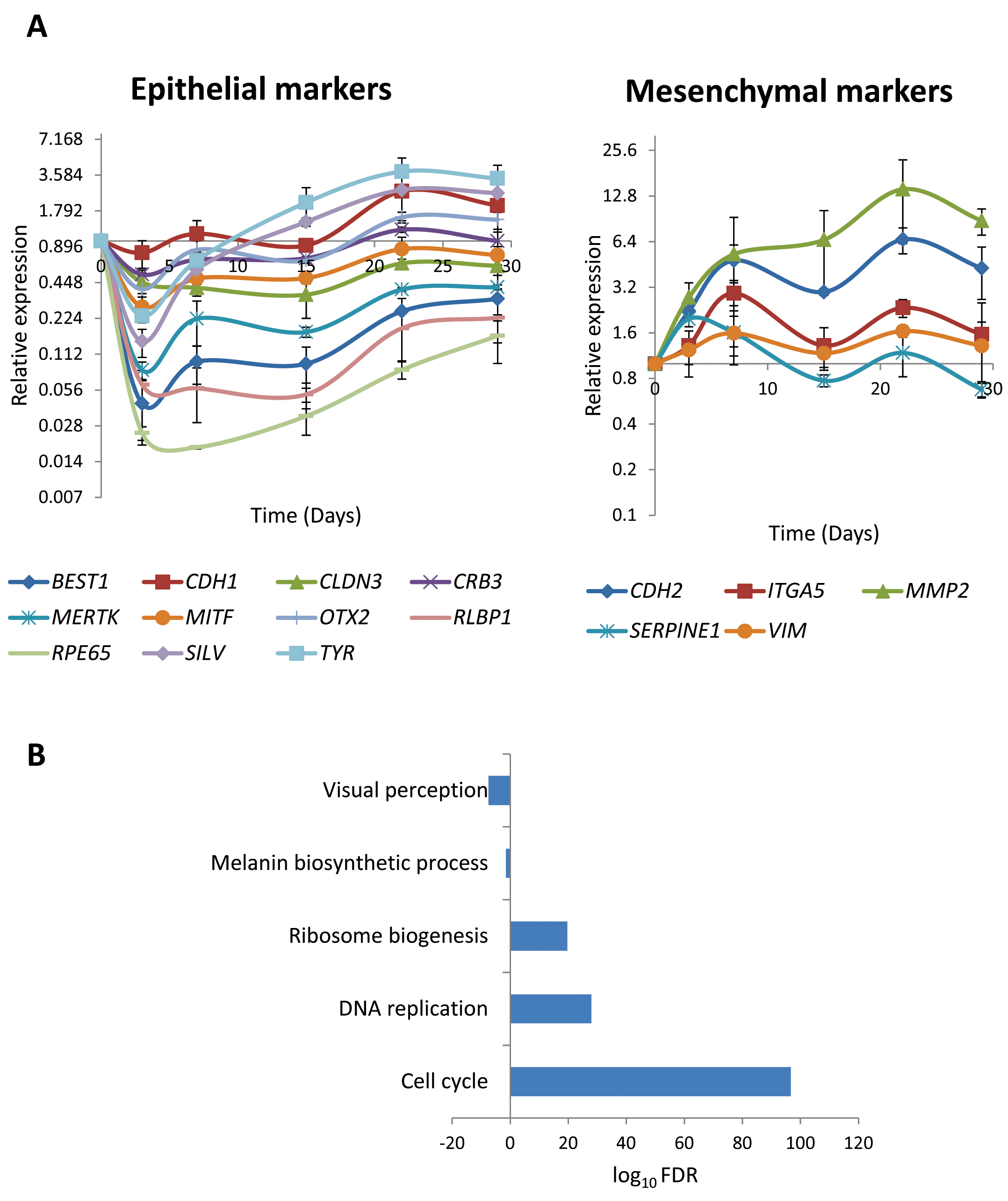

Supplement: S1 Fig — A. qPCR quantification of transcript expression (relative to Day 0) of epithelial and mesenchymal markers over a timecourse of RPE culture. ATP5B and CYC1 are used as housekeeping genes. Bars represent Mean ± SD (n = 3). B. Gene set test significance P values for exemplar GO terms for Day 3 versus Day 0 (P < 0.05 for all terms). High values indicate up-regulation at Day 3 relative to Day 0 and low values indicate down-regulation. (TIF) [file pone.0130379.s001.tif]

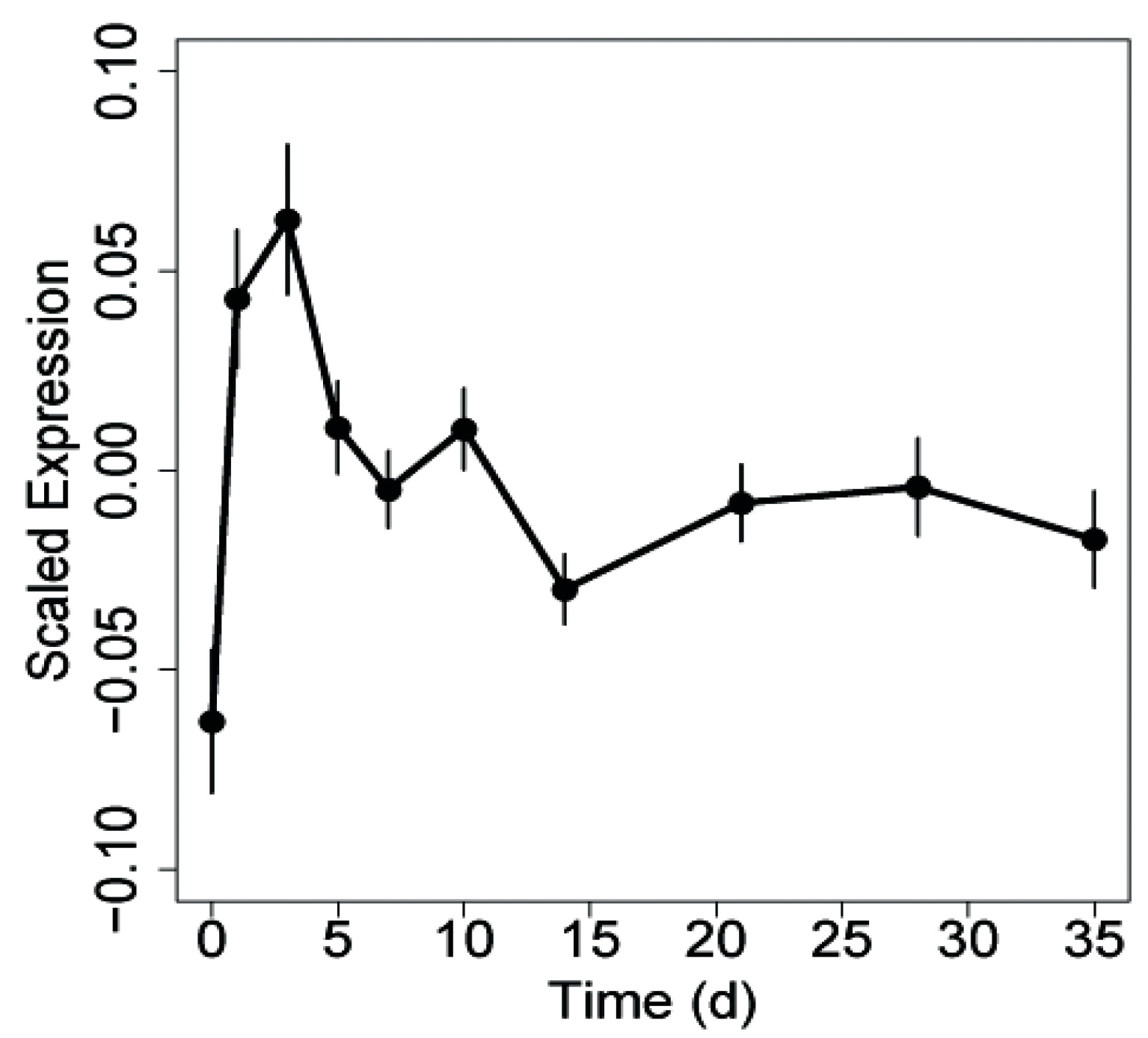

Supplement: S2 Fig — The raw microarray expression data of all genes containing FOXM1 peaks was mean centered and scaled to unit variance prior to plotting. Bars represent standard error. (TIF) [file pone.0130379.s002.tif]

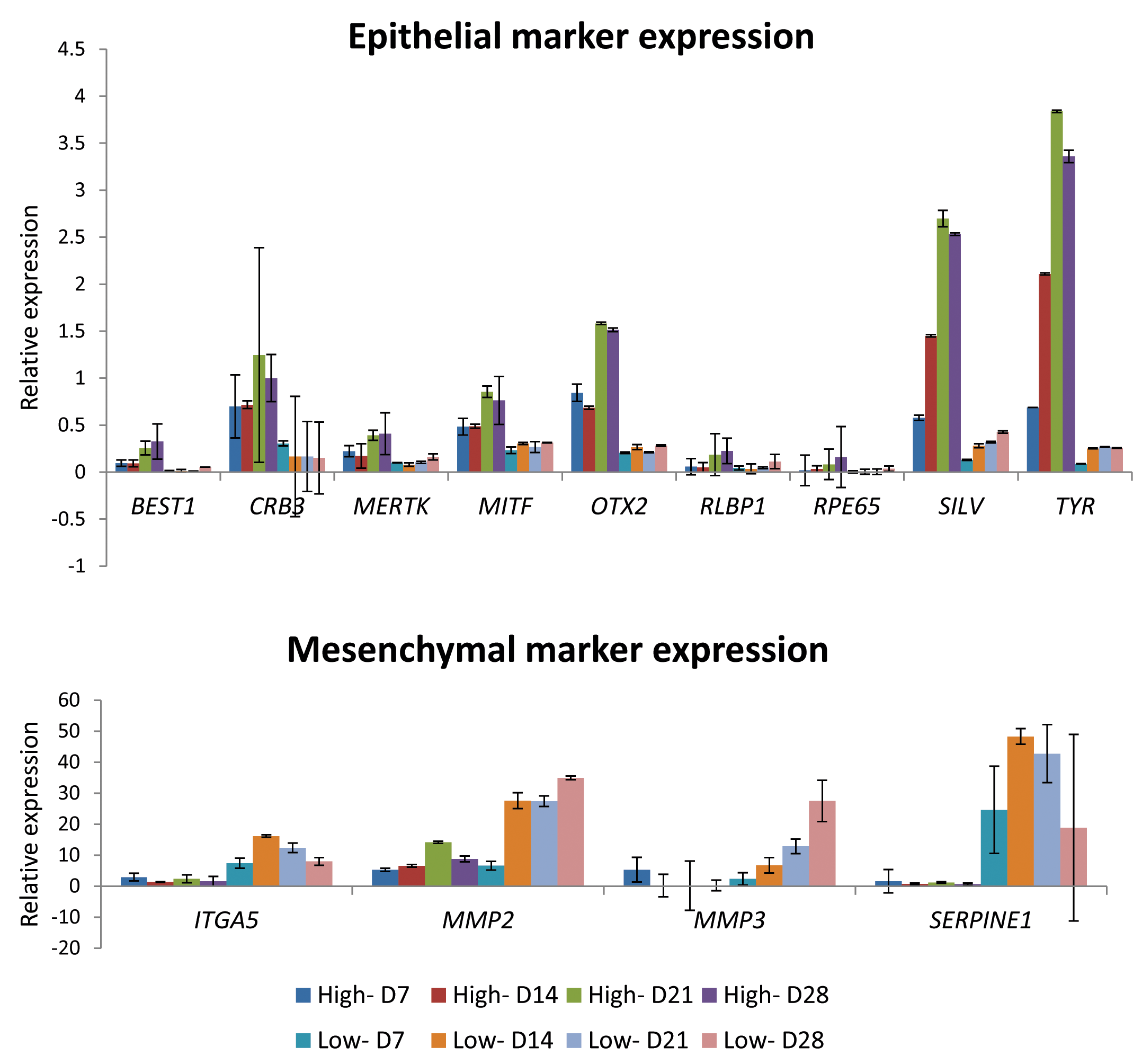

Supplement: S3 Fig — qPCR quantification of transcript expression (relative to Day 0) of epithelial (top) and mesenchymal markers (bottom) over a timecourse of RPE culture where cells are seeded either at high (100000 cells/cm2) or low (8000 cells/cm2) density. ATP5B and CYC1 are used as housekeeping genes. Bars represent Mean ± SD (n = 3). (TIF) [file pone.0130379.s003.tif]

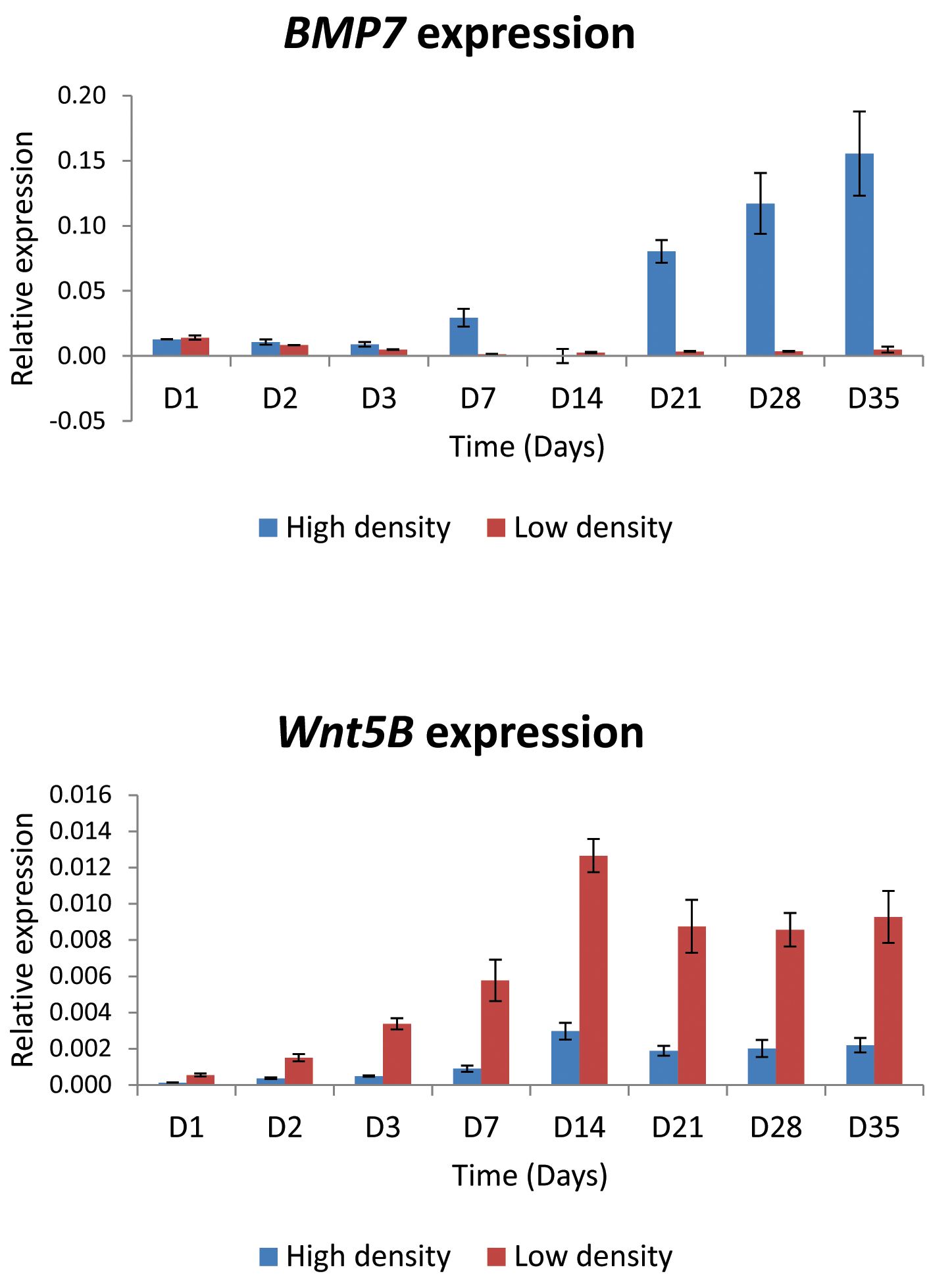

Supplement: S4 Fig — qPCR quantification of transcript expression of BMP7 (top) and Wnt5B (bottom) over a timecourse of RPE culture where cells are seeded either at high (100000 cells/cm2) or low (8000 cells/cm2) density. ACTB is used as a housekeeping gene. Bars represent Mean ± SD (n = 3). (TIF) [file pone.0130379.s004.tif]
